# Supplementary figures and images for: Towards optogenetic vision restoration with high resolution
Source: PLoS Comput Biol. 2020 Jul 15;16(7):e1007857. doi: 10.1371/journal.pcbi.1007857 (PMC7416966; doi:10.1371/journal.pcbi.1007857)

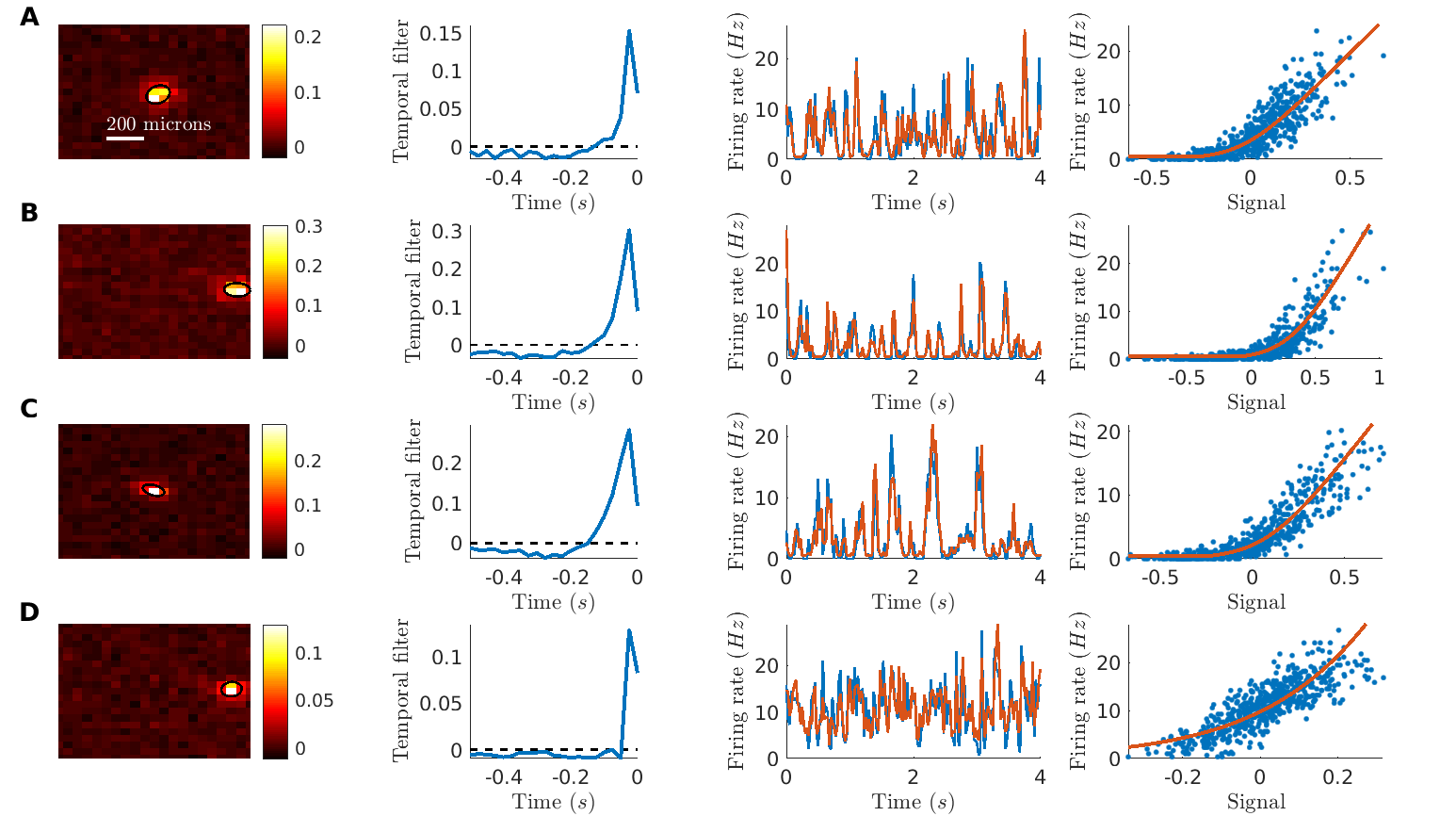

Supplement: S1 Fig — Left: Spatial and temporal receptive field. Center: experimental (blue) and predicted (red) firing rate in response to a repeated sequence of the checkerboard stimulus. Right: filtered stimulus plotted against the experimental firing rate for each time-bin of the repeated stimulus. Red: prediction of the firing rate using the non-linearity function. (TIFF) [file pcbi.1007857.s001.tiff]

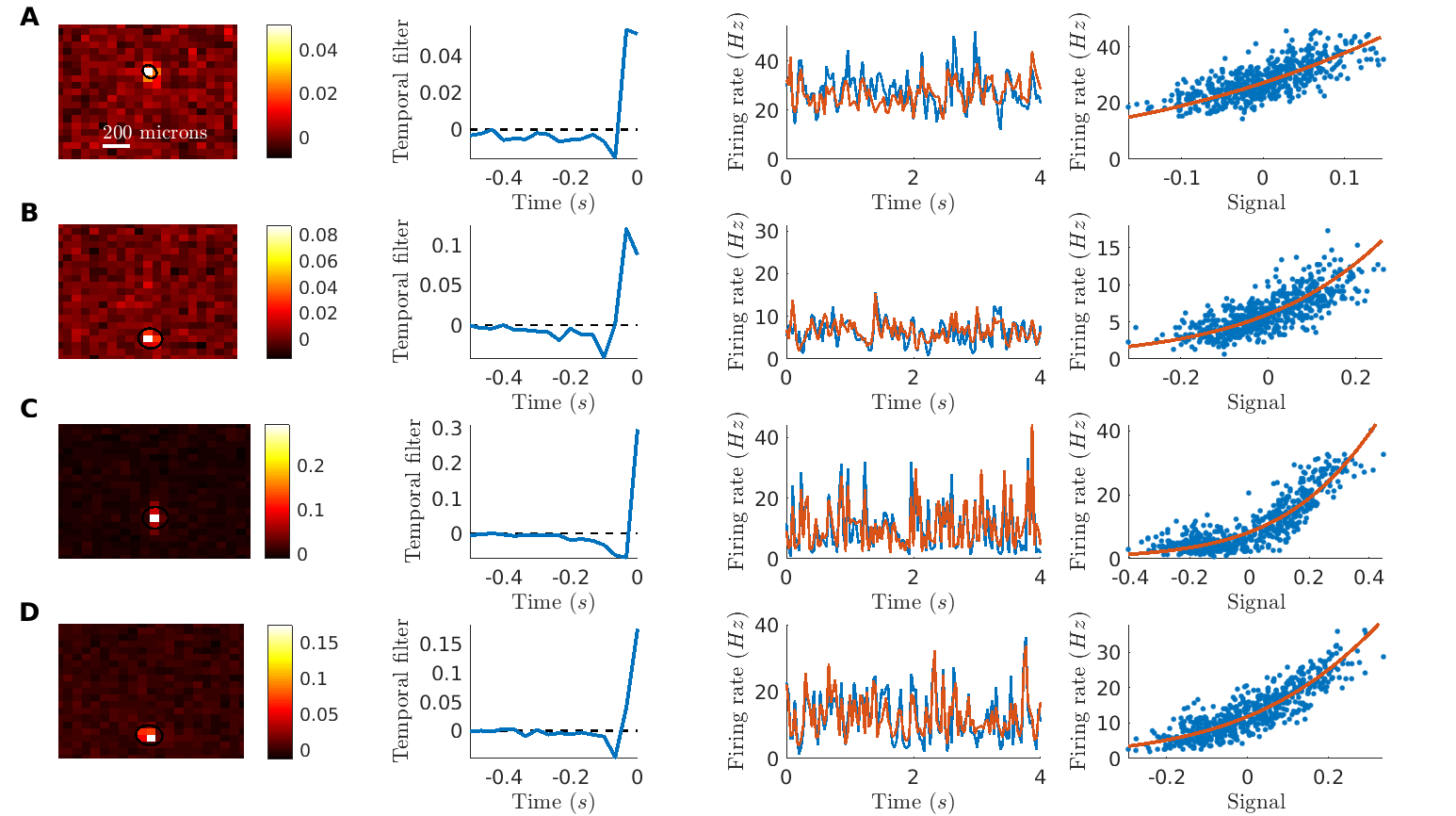

Supplement: S2 Fig — Left: Spatial and temporal receptive field. Center: experimental (blue) and predicted (red) firing rate in response to a repeated sequence of the checkerboard stimulus. Right: filtered stimulus plotted against the experimental firing rate for each time-bin of the repeated stimulus. Red: prediction of the firing rate using the non-linearity function. (TIFF) [file pcbi.1007857.s002.tiff]
