# Supplementary material for: Towards optogenetic vision restoration with high resolution
Source: PLoS Comput Biol. 2020 Jul 15;16(7):e1007857. doi: 10.1371/journal.pcbi.1007857 (PMC7416966; doi:10.1371/journal.pcbi.1007857)
Supplement: Attachment — Submitted filename: RefRep_acuity_PCB.pdf [file pcbi.1007857.s004.pdf]

In the following we address all the comments in detail. Text from the reviewers is in **black**, our replies are in **blue**, and the description of manuscript modifications is in **red**.

**REVIEWER 1**

*This manuscript is an interesting and fairly straightforward study on the receptive field size of ganglion cells in mouse and macaque retina that have been given photosensitivity using a variant of channel rhodopsin supplied by an AAV virus. The receptive field size was measured using a checkerboard stimulus to produce a spatio-temporal Spike-Triggered Average from recordings of isolated retinas using a MEA. A model of the transfected ganglion cell array was created from the average ganglion cell STA responses using a LN model that generated spikes with stochastic timing, placed on a square grid with spacing set from the density of transfected cells. The visual acuity was then predicted from Bayesian tests of the model's responses evoked by visual stimuli randomly jittered to simulate eye movements.*

*The manuscript is relevant to recent attempts to produce high-acuity vision for the blind using optogenetics. It seems appropriate for the journal because it is an excellent combination of electrophysiology and computational modeling. Overall it is well-written and the figures are adequate and appropriate. However, I have several major concerns about the methods and the resulting acuity derived from the model, and several minor suggestions about wording.*

We thank the referee for his/her positive comments and suggestions. Below we address all his/her concerns

*1. Receptive field size. As mentioned in the abstract and main text, looking at Figures 1 and 2, the reader sees no evidence of the axon within the optogenetic receptive field (RF). But the small RF diameter, modeled in Figure 3D, seems a different issue. The measurement of RF size with a 50 um check size in mouse and 67 um check size in macaque seems difficult to reconcile with the estimated size of ~93 um. With the assumption that the receptive fields were circular (and e.g. Gaussian), it might be reasonable to measure such an average RF diameter only 40% larger than the check size. But with a stationary grating, many RFs would have likely been misaligned with the borders of the checks so the STA average could not accurately reflect the actual diameter or shape. Without the circular Gaussian assumption, such a coarse check size seems inappropriate because it would not allow accurately defining RF shape and size. If the checkerboard stimulus could move (e.g. with some jitter, as in the model) one could imagine a higher resolution than the check size. But to ask a question about the shape of the RF, i.e. to what extent the ChR is in the axon and/or dendrites, the 67 um check size would seem too coarse to determine RF shape and size. Although the reader wonders about the significance of the exact RF size on acuity (see point 2 below), the measurement of RF size seems questionable. For the purpose of reporting the results on RF size, it would be more convincing to show several of the RFs with finer scale plots, or even better, measured with finer check size.*

We agree with the reviewer that the check size used here limits the precision of the receptive field size estimation, and that this does not allow us asking to what extent the optogenetic protein is present in dendrites and the part of the axon near the soma.
However, using a coarse check size probably leads to an overestimation of the receptive field size. Smaller receptive field sizes would lead to a better acuity. We have conducted additional analyses to estimate the uncertainty on the receptive field size. We found that this uncertainty is 7 microns (see Methods for the estimation). A change of the receptive field size of the order of 7 microns would have little effect on the resulting acuity (fig 3D).
We thus conclude that the coarse check size here should not affect our results on acuity estimation.
However, we wanted to make a different point here, related to an issue that appeared in other strategies to restore vision.
Several works aiming at restoring vision using retinal prosthesis showed that electrical stimulation by epiretinal implants, one of the leading solutions to restore blindness (see e.g. the ARGUS II implant), could activate distant ganglion cells, hundreds of microns away, through the stimulation of their en passant axons (Fried et al 2009, Weitz et al 2015). As a result, this means that a neuron can be activated by very distant electrodes, and that its "electrical receptive field" is very broad, covering several hundreds of microns. This is an important issue since several works have suggested that this distant activation of ganglion cells was the main limiting factor for the acuity reachable with retinal implants (Beyeler et al 2019, Weitz et al 2015).
Our aim was to test if a similar very broad receptive field could be found in our data, which would be a strong limiting factor for the resulting acuity. Our results show that, despite the experimental limitations, the receptive field size is much smaller than several hundreds of microns, i.e. what was found in the electrical receptive field found in previous studies, and that a similar activation of axons, activating ganglion cells over a broad area, is unlikely in our case.

We have added additional analyses about the uncertainty on the receptive field size (in Results and Methods), more examples of receptive fields (fig. S1 and S2), and acknowledged that the coarse check size limits our estimate of the receptive field size.

*2. The relation between receptive field size, cell spacing, and acuity is not well described. Large receptive fields, i.e. larger than the cell spacing, will obviously reduce effective acuity, but it is not clear how receptive fields much smaller than the cell spacing can improve acuity. Although Fig. 3D,E show acuity vs. RF diameter and acuity vs. transfection ratio (i.e. relative density), it is difficult for the reader to understand how acuity could be high (e.g. 20/40) with very small receptive fields (e.g. size of a soma, 10-20 um). Given a density of 51 cells/mm2 the average spacing in the real retina is ~140 um -- but with a transfection of 40%, the spacing of transfected cells is likely to be quite random and ~50% greater, ~220 um. With average spacing of 220 um it is difficult to imagine how receptive field sizes of less than 100 um, e.g. 10 - 50 um, could give improved performance in discriminating letter rotations.*

*The reader needs some explanation of how a receptive field the size of a cell's soma, in an array of cells spaced on average 220 um apart, could give higher acuity than one would predict from the spacing alone. It would seem that the term "hyper-acuity" should be applied in this case where the receptive fields are much smaller than the average spacing. Generally hyper-acuity described in the literature is only relevant for discriminating certain stimuli -- but usually not for letter rotations. It would help the naive reader to add a short section on hyper-acuity with appropriate references.*

*The main conclusion, that optogenetic vision could support useful high-resolution vision, seems based mainly on the lack of ChR sensitivity in the axon and the average cell spacing. The measurement of RF size and its relation to acuity seems less convincing.*

We apologize for the misunderstanding: the cell density is 51,108 cells/mm^2 (fifty one thousand one hundred eight) and not 51.108cells/mm^2. The comma should be read as a thousand separator, not a decimal one (see also point 10). This corresponds to an average inter-cell distance of around 4.4 microns (which is possible because in this foveal region, ganglion cells stack over several layers), and raises to about 10 microns when a 40% transfection ration is taken into account. This is why receptive field much smaller than 100 micron improve the performance in a discrimination task. For these reasons we do not think the term "hyper-acuity" should be applied here. We removed the comma as a possible source of confusion.

*3. Cell type. Although the issue of interpretation of the signals from ON vs. OFF cells by cortical circuitry is discussed, there appear to be other related issues that would be a problem in assessing the performance of the model. Since the cell types of the recorded cells have not been reported, and are likely unknown, the reader wonders whether large and small cells, e.g. magno vs. parvo types, or other types typically recorded in macaque, would be transfected -- and in what proportions. Different cell types are known to have different responses: e.g. transient vs. sustained -- and these if averaged would likely reduce the Bayesian performance. The question this raises is the relevance of the model with the assumption that different cell types are transfected and stimulated, and may project to different cortical areas that may not be able to directly integrate an acuity discrimination with each other. Therefore, it would seem that the acuity estimated is only an upper limit, and that the actual acuity from a real transfection would likely be substantially lower.*

*The issue of cell type also is relevant to the receptive field size, as it seems possible that different cell types could have different transfection patterns that would affect their receptive field sizes and shapes.*

The referee is right, in macaque different ganglion cell types have very different properties, including different morphologies, functions and projection targets. However, in our macaque experiments we recorded from the region near the fovea, and here most of the ganglion cell are

midget cells (Dacey 1994). We expect that this population is therefore quite homogeneous. The main heterogeneity here should be the division between ON and OFF cells.

We agree that our model assumes that the brain can make the best use of the ganglion cell activity, but, because different types project to different areas, this assumption might not hold, and as a result the resulting acuity could be lower.

There are several papers showing that the brain might not be able to make the best use of all the signals coming from the different cell types (Smeds et al 2019, Schiller et al 1986). However, recent works (eg Rompani et al 2017) have shown that, in fact, neurons might integrate the output of several cell types together, as early as in the LGN. It is therefore unclear if one should only take into account the output of a single cell type, or if the brain can pool the results from several cell types together.
This point is thus far from being resolved in the literature. We have discussed these points more extensively in the discussion, and we have added details about the transfected region in the results. We have also acknowledged more clearly that our results give an upper bound on the resolution.

We added further details in results and discussion sections.

*4. Grid geometry. It would seem that a semi-random grid geometry, set with the average nearest-neighbor distance and regularity from the real transfection pattern, instead of a square grid as contained in the model, would be more appropriate -- as then the model would more closely reflect the pattern of spacing of the real cells and possible hyper-acuity.*

In order to account for irregularities in the cell mosaic, we performed a second acuity simulation where the spatial locations of the RGCs have been jittered by adding a Gaussian noise (zero mean and 3micron std, to be compared with the 4.4 microns average inter-cell distance) to both X and Y coordinate.

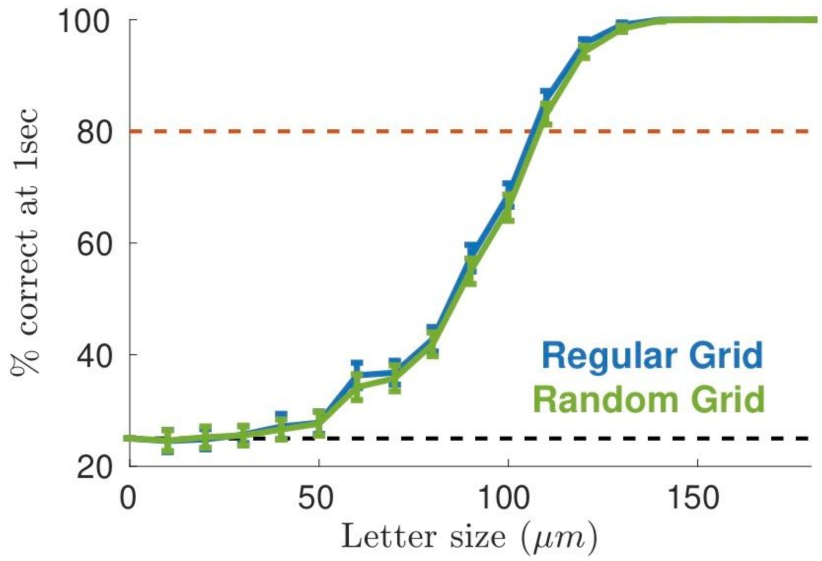


*Percentage of letters decoded correctly after a 1s exposure, against the letter size for both regular and random grid of cells.The dashed line is the 80% accuracy limit that we used in our definition of acuity*

As shown in the figure above, the resulting acuity is only slightly different, and within the expected statistical error: intercell distance is much smaller than receptive fields, and adding some noise does not significantly affect the results.

*5. l 80, "Details ... has been detailed", suggest "Details ... have been detailed"*
Thanks for this, corrected

*6. l 113, "due to photoreceptor", suggest "from photoreceptors"*
Thanks for this, corrected

*7. l 113, "We performed a similar experiment than for mouse", suggest "We performed a similar experiment as for the mouse".*
Thanks for this, corrected

*8. l 116, "we could still find STA with a well-defined receptive field in some ganglion cells (Fig 2A,B) with an average diameter of 92.8+/- 10 um", suggest "in some ganglion cells we could still find a STA with a well-defined receptive field (Fig 2A,B) with an average diameter of 93 +/- 10 um".*
Thanks for this, corrected

*9. l 133, "were placed on a squared grid", suggest "were placed on a square grid".*
Thanks for this, corrected

*10. l 137, "51,108 cells/mm2", suggest "51.1 cells/mm2". Although a comma is often used as a decimal point, for this journal a dot "." seems more appropriate.*
This is probably the source of confusion that raised the remarks of point 2. Here the comma is not a decimal separator but a thousands one, according to both USA and UK conventions ( https://docs.oracle.com/cd/E19455-01/806-0169/overview-9/index.html ). Cell density is 51.1 thousands cells for square millimeter. We removed the comma as a possible source of confusion.

*11. l 147, "Using this decoder is equivalent to assume that", suggest "Use of this decoder is equivalent to the assumption that".*
Thanks for this, corrected

*12. l 308, "and compute the mean response", suggest "and computed the mean response".*
Thanks for this, corrected

*13. l 158-189 "these 110 microns corresponds", suggest "these 110 microns correspond".*
Thanks for this, corrected

*14. l 308 "and compute the mean response", suggest "and computed the mean response".*

Thanks for this, corrected

*15. l 326, "only the center of the cells", suggest "only the center position of the cells".*
Thanks for this, corrected

*16. l 330, suggest placing "Deny et al." inside parentheses: "(Deny et al., 2017)".*
Thanks for this, corrected

*17. l 337 "is to mimic", suggest "was to mimic".*
Thanks for this, corrected

*18. l 339, A white letter on a black background is generally not considered to be 100% contrast, as the overall level of illumination would depend on the fraction of the visual field that is covered with white. In real experiments, the background level must be measured and reported, as it sets the adaptation level in the visual system. A truly black background would dark-adapt the retina and therefore the white letter stimuli would generate an undetermined level of illumination, an undetermined level of adaptation, and undetermined contrast. This effect might not affect the results from the model -- but it should be described in the text so as not to confuse the reader.*

We agree that this was confusing. Here the ganglion cells are activated thanks to an optogenetic protein, and in this case low light levels should not trigger any adaptation, since it will not be high enough to activate the optogenetic protein. We have removed the reference to contrast and have added clarifications in the text.

**REVIEWER 2**

*The manuscript by Ferrari et al. measures the receptive fields of optogenetically transdued retinal ganglion cells in mouse retina and in primate retina. Based on the spatial extend of the estimated receptive fields the authors build a model and theoretically estimate spatial acuity. The manuscript adresses a highly relevant topic, but the authors should give the paper the academic depth it deserves.*

We thank the referee for his/her positive comments and suggestions. Below we address all his/her concerns

*In the current version I cannot recommend the manuscript for publication. Please find my arguments below:*

1. *In the abstract the authors state: „..the spatial resolution of optogenetically-reactivated retinas has not been measured with fine-grained stimulation patterns."*

*This statement is not correct, see i.e. the recent publications by (a) Barrett et al (2016 / https://www.nature.com/articles/srep33565) and by (b) Ganjawala TH et al. (2019) ( https://www.ncbi.nlm.nih.gov/pubmed/31010741 ) . These studies investigated mouse retina only; however, the spatial resolution was measured in very great detail. This experimental scientific rigour is missing in the manuscript by Ferrari et al.*

The referee is right, we should have cited these works. However, as mentioned by the reviewer itself, our work adds several contributions to these studies, which we think are important:

1) As mentioned by the reviewer, we present results on macaque retina, and not only on mouse. This is an important difference because macaques have a fovea, i.e. a region of high resolution, unlike mice. For this reason, predicting the acuity based on results obtained on mouse seems incomplete, and data on primate is a significant contribution.

2) A point that was unclear in our manuscript, which we have tried to clarify and develop in the revised version of this manuscript, is that the connection between the responses of neurons, and the information available for the brain to form percepts, is far from trivial, and our study aims at making a rigorous connection between them through a model of the whole population of ganglion cells.

Many works, including the work from Shadlen, Newsome and colleagues (e.g. Zohary et al, Nature 1994), have shown that care should be taken when comparing the sensitivity of single neurons, and the perceptual sensitivity. They demonstrated that, in general, there is no straightforward link between one and the other (see also Shapley and Victor, Science 1986, showing a case where single ganglion cells were more sensitive than perception). These studies also established how modeling can be used to connect neural responses and perception, by building a model of the entire neural population responding to the stimulus, and quantifying how much information can be read out of this population. This is exactly the strategy we are following here, while previous studies like Ganjawala et al (2019) only characterized single neuron sensitivity.

Note that there is a specific interest in doing this modeling of the population in the case of ganglion cells reactivated by optogenetics because we could find a simple model that predicts well ganglion cell responses to stimulation patterns, something which would be much more challenging with a normal retina.

Barret et al (2016) also tried a direct decoding of neural population activity. However, it is important to note that only part of the full population is recorded: if one wants to estimate the sensitivity of the full population of retinal ganglion cells, which is what the brain can access, modeling is necessary to avoid missing information provided by cells that were not recorded during the experiment (see also our reply below about the comparison with this specific work).

To conclude, while the studies mentioned by the reviewer are definitely interesting and worth citing, they do not build a model of the full population, something that seems necessary to

understand the resulting perception. Finally, they present data recorded in the rodent retina, while we also present data from macaque retina.

In the new version of the paper, we now cite and discuss these works, clarify our strategy and compare it to these previous works.

2. *The authors measure the spatio-temporal receptive field of optogenetically transduced retinal ganglion cells using white-noise checkerboard stimuli.*

*This approach is not new either and has been reported recently by Pisano et al. (2017) https://www.nature.com/articles/s41598-017-15741-7. The authors should (a) cite this previous work and (b) explain if their results confirm / reject this (and pontetially other) work.*

The referee is right, Pisano et al. (2017) used checkerboard stimulation and STA analysis to measure receptive fields in optogenetically transduced cells. However,
1) they didn't link these measurements to resolution and acuity. We do not claim that using a checkerboard to measure receptive field is novel, this has been used for decades. Our main point is to relate this to an estimate of acuity using a modeling of the full population, a point not addressed by Pisano et al (2017).
2) we used AAV to transduce cells, whereas they used mouse lines, which is not a viable option for therapeutic application to human patients. This difference may provide different results: the expression level of the optogenetic protein might be different. For example, there could be more or less expression in the axon depending on the genetic strategy used to express the protein.

We now cite and discuss Pisano et al. (2017) in the introduction.

*Furthermore, I am concerned that the temporal time course of the receptive field (Fig 1B) is wrong. In line 89 the authors state „ „The latency of the peak response was around 25 ms.“ This value is about ten times longer than direct activation of retinal ganglion cells in the mouse retina ! The peak response latency is not reported fort he trace presented in Fig. 2B.*

We apologize if our explanations were not clear enough. We measured the temporal course of STA with stimuli project at 40Hz and 30Hz for, respectively, mouse and macaque. These frequencies set the limit of our temporal resolution in the estimation of the peak-response latency. For mouse we found the peak at the second time-bin, meaning that the latency is between 25 and 50ms. However, please also note that the temporal trace is above zero for the first time-bin, at about one third of the peak. This means that the onset of the response start between 0 and 25ms, in line with what the reviewer says, even if the maximal activation is between 25 and 50ms. For the macaque, we found in most of the case the peak-response in the first time-bin, i.e. a latency smaller than 33.3ms, and some times in the second, i.e. a latency smaller than 66.6ms.
We added more explanation and details in the result section.

3. *The authors base their analysis for macaque retinal ganglion cells on 3 cells, i.e. those thre cells which already showed a high response relaibility ! This is a very low number and cetainly not enough for statistical comparison. Please include all 9 RGCs (line 303) recorded in the macaque retina.*

Thanks to the new experiment we now include 15 cells (out of 25) instead of 3. However, we still prefer to keep the reliability threshold because this assures us to consider only well-transfected cells. Note that in our acuity estimation we account for transfection issues by considering that only 40% of the cell are efficiently transfected: this means that we estimate that the ratio of high reliability cells is 40%, based on a previous study counting fluorescent cells (Chaffiol et al).

4. *The nonlinearity oft he LN model may be shown as well. It would be instructive to see if the the prediction performance (Fig. 1C&D; Fig. 2C&D) is different for linear estimates as compared tot he LN model.*

Strictly speaking, a fully-linear model could generate negative firing rate, and thus cannot be used to predict ganglion cell responses. However, as non-linearity we are using a soft-plus (smooth ReLU) with the good offset and threshold, and this is almost equivalent to a linear model where negative inputs are set to zero.
We added plots of the non-linearities for both mouse and macaque in the supplementary.

5. *The performance of predicting the stimulated response based on the LN model is not well explained. Please provide additional details (i.e. was there any temporal binning performed and if so at what resolution ?).*

We thank the referee for pointing out this lack of clarity in our explanation. Yes, both the model prediction and the PSTHs were binned with temporal windows locked to the frame update (30Hz for macaque and 40Hz for mouse). We added these details in the corresponding method section.

6. *In the last part of the manuscript the authors build a model and use a Baysian decoder to predict the orientation of a presented stimulus („letter E“). Again, baysian decoding is not new and has been performed for optogeentically transduced retinal ganglion cells in the work of Barrett et al (2016 / https://www.nature.com/articles/srep33565). The in silico approach in this manuscript might be different but this difference needs to be explained and discussed in detail.*

As explained above, one of the main differences between the approach of Barrett et al (2016) and our approach is that we use modeling to simulate how the full population of ganglion cells responds to the stimulus, while they perform decoding based on the neurons they recorded.

On one hand, direct decoding of the recorded population, like in Barrett et al (2016), has the drawback of missing the activity of the cells that are not recorded. This can be an important issue when trying to estimate the resolution of the full reactivated retina. On the other hand, direct decoding allows skipping the use of a model. If the model is wrong, it could introduce errors in the estimate of the resolution. We have tested the performance of our model and shown that it predicts ganglion cell responses well. We thus think that the resolution derived from our model is accurate.

Another important difference between the two approaches is the ensemble of possible stimuli when decoding the retinal responses. In the case of Barrett et al, the possible stimuli are the different letters presented. In our case, following Burak et al (2010), we also had the position of the letter as an additional variable, unknown to the decoder. At each time step, the position could change, mimicking the impact of eye movement (an approach also used in Burak et al). As a result, the possible stimuli are thus all the possible letters in all the possible locations. This is a very large ensemble, and to infer which letter was presented, one need to also average the posterior over the possible positions in our Bayesian decoding. This is a more challenging problem, but closer the problem the brain needs to solve when only accessing the ganglion cell activity, not knowing where the letter is a priori because of fixational eye movements.

We have added these clarifications in the text.

*bstract: it is academically shallow and reads more like a commercial motivator. Though it is no problem that about half of the authors are potentially conflicted with industry, they should apply the same rigor to the abstract that they would to their other research publications. It currently reads as if written by a PR-agency than by scientists. As it stands now, the abstract describes how novel the technique is and how much work went into it. But the abstract is missing reference to any statements regarding efficacy, safety, current challenges and future directions or a carefully balanced discussion about benefits and risks.*

We think the space constraint for the abstract do not allow a complete discussion between benefits and risks. However, we have modified the abstract to match the reviewer expectations.

*The interested reader wishes to know, for example, how many patients have benefits and what kind of benefits one can expect, for which cases it is useful, the long term consequence, and possible surgical or other risks.*

This is of course a complex issue that cannot be contained in the abstract. We have mentioned these points in the discussion.

*Minor revisions:*

1. *Figure Caption 1D: „ Prediction performance of the model against reliability of the response (see methods).*

*I do not see such a plot. Instead it seems as though average values are presented. Could you plot for each cell the reliability (x-axis) against prediction performance ? It would be informative to check for linearlity.*

We thank the referee for pointing out the lack of this information. We added a scatterplot reliability Vs performances, with a point for each cell.

2. *The visual stimulus is poorly described. I checked the cited reference (Deny et al. 2017), where exactly the same sentence is provided. „The stimulus was displayed using a Digital Mirror Device and focused on the photoreceptor plane using standard optics." Please provide the DMD supplier company and describe what optics has been used (microscope ?)*

We thank the referee for pointing out the lack of this information. The DMD supplier was ViALUX and the optic was a microscope. We added this information in the method section.

3. *Line 168 – 171.The authors claim that no axonal stimulation occurs. This may be true on average (spike-triggered responses) but cannot be excluded by this approach. Additionally the authors seem to use only one light intensity, which could be below the axonal stimulation threshold. Please be more specific here.*

The referee is right, we can not exclude possible axon stimulation at larger intensities. Yet, our results show that there is an intensity range at which cells, but not axons, respond. We added this further explanation in the discussion.

**REVIEWER 3**

*In their manuscript 'Towards optogenetic vision restoration with high resolution', the authors address an interesting topic. The analysis is currently quite limited. Especially, given the complexity and value of the primate study, the authors should present their data in a less qualitative way. The authors should be able to improve the value of their study significantly without the need for additional experiments.*

We thank the referee for his/her positive comments and suggestions. Below we address all his/her concerns

*The paper is to a great extent motivated by the fact that the whole cell becomes light-sensitive. However, this is hardly quantified in the manuscript. The variance of a Gaussian fit does not*

*represent the possible influence of a light-sensitive axon appropriately. In figure 1A some sensitivity is visible at 11 o'clock. Is this a common observation? Is this in the direction of the axons? Is the SNR of the measurements sufficient to see axons? One could imagine e.g. an estimation based on the volume of the soma and axon, and the size of the stimulus grid.*

The sensitivity at 11 o'clock it's probably a random fluctuation of noise as it is not apparent in the RF of any other cells (Added in supplementary). Unfortunately, for this specific cell, we were not able to relate it with the axon position.

We would like to clarify our claim here: we do not relate precisely the receptive field with the detailed anatomy of the neuron. The point we want to emphasize here is that we did not find that a distant stimulation that activates the ganglion cell. This would have happened if stimulating the axon even far from the soma would be enough to evoke a response. In that case we would have had a very broad, elongated receptive field. This is not a purely theoretical possibility: it happened exactly for stimulation with electrical prosthesis. Here we show this is not the case. Of course this does not exclude that the part of the axon closest to the soma is light sensitive.

We added this further explanation in the discussion. See also answer to referee-2's comment 3.

*The measured receptive field diameters are much larger than those of the somas'. How strong is the influence of the stimulus grid? If the primary dendrites play a significant role, a significant variation across cell-types might be observable. Are microscopic images of the cells available? In the primate, a significant number of cells should be midgets and parasol cells with distinct differences. Do all RGCs share a similar time course?*

The referee is right, as we mention in the discussion, primary dendrites may play a role in the activation of ganglion cells, and this could potentially lead to different spatial properties in the receptive field, depending on the cell type. However, we do not observe such differences. In particular, all the recorded RGCs share a similar time course.

This can be explained by two reasons that are not mutually exclusive: first, as the reviewer mentioned, the size of stimulus grid may be too coarse to distinguish different cell types. This coarse check size was necessary to maximize the Signal to Noise Ratio of ganglion cell responses, but limits the precision of the receptive field size estimation. We have added an estimation of the uncertainty in receptive field size estimation in our results and methods.
Second, because the transfected cell are all in the foveal ring, most, if not all the recorded cells are midget (Dacey, 1994). In one of the macaque retina recorded we also imaged the transfected cells in a previous work (Chaffiol et al, 2017). However these cells were densely packed (see figure), and it is therefore hard to tell if there is more than one type here. Based on the literature, our conclusion is thus that most cells must be midget cells, but we cannot demonstrate this directly.
We have added these explanations in the text.

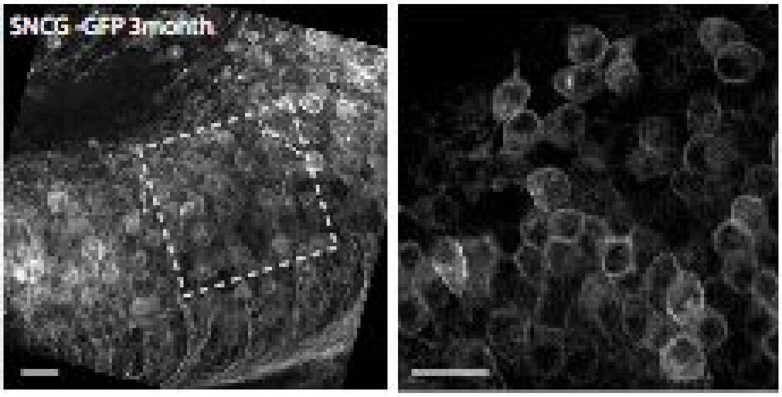


*Two-photon images of the peri-foveolar region of NHP, injected with AAV2-hCatCh-GFP displaying the high density of transfected cells with membrane-bound expression. The white dotted square on the left indicates the area displayed at higher magnification on the right. Reprinted from Chaffiol et al 2017*

*'...which correspond to ganglion cells where the expression level of the optogenetic protein was high enough.' Please provide quantification.*

We apologize for the lack of clarity here. Our recordings were performed over a large area of the retina, and the foveal ring was the only part of it where the optogenetic protein was expressed (see figure below, reprinted from Chaffiol et al 2017). Expression was restricted to the perifoveal ring due to limitation in the used AAV. This explains why many recorded cells did not show optogenetic responses. As a side note, the criterion we used to select cells with a strong modulation was already described in the methods (reliability score above 0.5).

We removed the sentence, but explained in more details in the methods how the expression of the optogenetic protein was achieved.

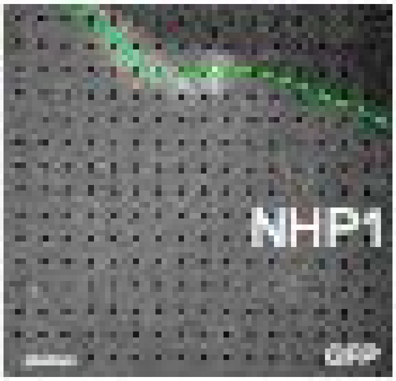


*The macular area is indicated by dashed green lines and the black dots represent the locations of the MEA recording electrodes. Reprinted from Chaffiol et al 2017*

*'The small ratio in the macaque is due to the fact that many recorded neurons were outside of the foveal ring, and therefore not transfected efficiently by the AAV...' Can this be quantified?*

What can be estimated is the number of electrodes overlapping with the foveal ring, when GFP is co-expressed with the optogenetic protein, so that the transfected area can be visualized. In

one experiment where this was the case, we estimated that to be around 35 electrodes, out of 252.
We have reported this in the text.

*Please provide evidence that the untreated tissue showed no light responses.*

We have shown this in a previous paper (Sengupta et al 2016).
We have added this point and a reference to this paper in the text.

*'Assuming that discriminating a letter of 25 µm gives a 20/20 acuity'. Please provide citations to go from the macaque to the human.*

Ganglion cells in the fovea of macaque and human retinas have approximately the same size, and comparable morphology (Dacey and Petersen, 1992). We therefore assumed that receptive field size as measured here would not change dramatically when going from macaque to human, and therefore that the resolution enabled by these transfected ganglion cells would not be changed when going from macaque to human.
We have clarified this in the text.

*'The letter was white on a black background (100% contrast)' A lower contrast might be a more realistic assumption.*

We apologize for the confusion here. In any optogenetic therapy, the patient will have to wear goggles that will amplify light intensity so that optogenetic protein can be activated (the amount of light required for this is much higher than ambient light levels). Stimuli will therefore have a much higher luminance and contrast than in natural environments.
We have clarified this in the text.

*'...the other ones were supposed to send no information about the stimulus and therefore removed' They should have a spontaneous background rate, possibly even a correlated firing pattern.*

The referee is right, these cells have a spontaneous activity. Yet, our optimal Bayesian decoder will not be affected as their firing rate will be stimulus-independent.
We added a comment in the methods.

*'...flashed the letter E at a new random position every 67 ms' Fig 3B should be labeled 'repetitions' or the model should use a continuous random walk as stimulus input.*

We have chosen to label the x-axis as 'time' because the change in position of the letter mimics fixational eye movements and we computed acuity as the performance after 1 second. Yet the referee is right in pointing out that we need to be more explicit. We added some more explanation in the main text and caption of figure 3.

*Please extend your discussion of the fact that the model is close to the best-case scenario. Possibly, tone down the abstract as this study analyses three cells and extrapolates them to 50000/mm2.*

Thanks to a new experiment on the macaque retina, we now include 15 cells with good SNR (out of 25) instead of 3 (out of 9). We consistently modified the abstract and the discussion.

*How many separate recordings are part of this paper?*

Three: one for the mouse and now two for the macaque.

*Is data used in other publications?*

The checkerboard responses have not been presented elsewhere, but other recordings of the same tissue have been published before, and not included here.

*What data is shared?*

We agree to share all the data after acceptance.

*Overall, please provide more quantifications and citations throughout the manuscript.*

We have added 14 more citations, 8 new figure panels, 2 supplementary figures of 16 panels each, and a number of new quantifications. We hope that the revised version of the manuscript satisfies the reviewer.

*'Here we developed an novel computational approach...' The computational approach is not new.*

Yes, the referee is right, the LN model and the ideal Bayesian analysis are not novel from a machine learning point of view, but nobody applied them to optogenetic therapy, vision restoration and acuity estimation on macaque retina. We have clarified this in the text.

REFERENCES

- Barrett, J. M., Hilgen, G., and Sernagor, E. (2016). Dampening spontaneous activity improves the light sensitivity and spatial acuity of optogenetic retinal prosthetic responses. Scientific reports, 6:33565.

- Beyeler, M., Nanduri, D., Weiland, J. D., Rokem, A., Boynton, G. M., and Fine, I. (2019). A model of ganglion axon pathways accounts for percepts elicited by retinal implants. Scientific Reports, 9(1):9199.

- Burak, Y., Rokni, U., Meister, M., and Sompolinsky, H. (2010). Bayesian model of dynamic image stabilization in the visual system. Proceedings of the National Academy of Sciences, 107(45):19525–19530.

- Chaffiol, A., Caplette, R., Jaillard, C., Brazhnikova, E., Desrosiers, M., Dubus, E., Duhamel, L., Mac ́e, E., Marre, O., Benoit, P., Hantraye, P., Bemelmans, A. P., Bamberg, E., Duebel, J., Sahel, J. A., Picaud, S., and Dalkara, D. (2017). A New Promoter Allows Optogenetic Vision Restoration with Enhanced Sensitivity in Macaque Retina. Molecular Therapy.

- Dacey, D. M. (2008). Physiology, morphology and spatial densities of identified ganglion cell. In Ciba Found. Symp, volume 765, page 12.

- Dacey, D. M. and Petersen, M. R. (1992). Dendritic field size and morphology of midget and parasol ganglion cells of the human retina. Proceedings of the National Academy of sciences, 89(20):9666–9670.

- Fried, S. I., Lasker, A. C., Desai, N. J., Eddington, D. K., and Rizzo 3rd, J. F. (2009). Axonal sodium-channel bands shape the response to electric stimulation in retinal ganglion cells. Journal of neurophysiology, 101(4):1972–1987.

- Ganjawala, T. H., Lu, Q., Fenner, M. D., Abrams, G. W., and Pan, Z.-H. (2019). Improved cochr variants restore visual acuity and contrast sensitivity in a mouse model of blindness under ambient light conditions. Molecular Therapy, 27(6):1195–1205.

- Pisano, F., Zampaglione, E., McAlinden, N., Roebber, J., Dawson, M. D., Mathieson, K., and Sher, A. (2017). Large scale matching of function to the genetic identity of retinal ganglion cells. Scientific reports, 7(1):15395.

- Rompani, S. B., Muellner, F. E., Wanner, A., Zhang, C., Roth, C. N., Yonehara, K., and Roska, B. (2017). Different modes of visual integration in the lateral geniculate nucleus revealed by single-cell-initiated transsynaptic tracing. Neuron, 93(4):767–776.

- Schiller, P. H., Sandell, J. H., and Maunsell, J. H. (1986). Functions of the on and off channels of the visual system. Nature, 322(6082):824–825.

- Shapley, R. and Victor, J. (1986). Hyperacuity in cat retinal ganglion cells. Science, 231(4741):999–1002.

- Smeds, L., Takeshita, D., Turunen, T., Tiihonen, J., West ¨o, J., Martyniuk, N., Sepp ¨anen, A., and Ala-Laurila, P. (2019). Paradoxical rules of spike train decoding revealed at the sensitivity limit of vision. Neuron, 104(3):576–587.

- Weitz, A. C., Nanduri, D., Behrend, M. R., Gonzalez-Calle, A., Greenberg, R. J., Humayun, M. S., Chow, R. H., and Weiland, J. D. (2015). Improving the spatial resolution of epiretinal implants by increasing stimulus pulse duration. Science translational medicine, 7(318):318ra203–318ra203.

- Zohary, E., Shadlen, M. N., and Newsome, W. T. (1994). Correlated neuronal discharge rate and its implications for psychophysical performance. Nature, 370(6485):140.
