# Supplementary material for: Towards optogenetic vision restoration with high resolution
Source: PLoS Comput Biol. 2020 Jul 15;16(7):e1007857. doi: 10.1371/journal.pcbi.1007857 (PMC7416966; doi:10.1371/journal.pcbi.1007857)
Supplement: Attachment — Submitted filename: Re_Re_Acuity.pdf [file pcbi.1007857.s005.pdf]

We thank the editor and the reviewers for the careful comments on our manuscript.

In the following we address all their comments. Reply to their comments are in **blue**, reference to changes in the manuscript are in **red**.

On the behalf of the authors,
Your sincerely
Olivier Marre

Reviewer #1: Review of Ferrari et al. (2019) "Towards optogenetic vision restoration with high resolution", PCOMPBIOL-D-19-00958_R1.

The authors have responded well to most of the reviewers' comments, as their revision has added additional information that clarifies many of the problems that had been identified. I only have minor suggestions for revision.

1. line 18, "might vary upon changing therapeutic strategy", suggest
"might vary with changes in the therapeutic strategy", or
"might vary upon changing the therapeutic strategy", or
"might vary depending on the therapeutic strategy".

Corrected, thanks.

2. line 20, "under conditions that our model helps determining.", suggest
"under conditions that our model helps to determine."

Corrected, thanks.

3. lines 89-90, Again I would suggest that the method used could not determine the receptive fields with the accuracy described. The problem as previously explained is the shape of the receptive field assumed in fitting the diameter.

We added "under the hypothesis of Gaussian shape".

Suggest changing "98.7+/-7" to "99+/-7", or even "100+/-7".

Modified into 99+/-7.

Suggest adding a comment about the "individual uncertainty of 3 micron," to

explain how this accuracy was determined based on the 50 um check size, and add a short section detailing the assumptions about the receptive field shape. In Methods a section (lines 394-398) mentions the use of a fitted two-dimensional Gaussian, but it would be helpful to explain why this would be appropriate for the image of a soma and proximal dendrites labeled by the AAV virus, and to explain the expected error by assuming a Gaussian shape..

As explained in the methods, we estimate the individual RF size using a bootstrap technique and under the hypothesis of Gaussian RFs. We agree with the reviewer that this approach might under-estimate these errors if the receptive fields deviate significantly from the Gaussian assumption, something that did not appear in our data. We agree that this lack of deviation might be due to the relatively large check size used in the recordings, but is in line with previous works on macaque ganglion cell receptive fields (Gauthier et al, 2009). Finally, when building the model, note that what accounts mostly for the SEM of the mean population RF is the dispersion across the population and not the individual uncertainties. Even accounting for this under-estimate would not change significantly this final SEM value. We have rephrased the corresponding parts of the results and added more explanations in the methods (lines 401-408).

Suggest change "of 3 micron" to "of 3 microns".

Corrected, thanks.

These revisions will not affect the manuscript's conclusion, but would be less confusing to the naive reader who might wonder how such an accuracy could be given with a check size of 50 um.

We thank the referee for these suggestions for improving our manuscript.

4. line 96, change "start" to "starts".

Corrected, thanks.

5. line 96, "in line with a direct light-activation", suggest "consistent with a direct light-activation".

Corrected, thanks.

6. line 121, change "photoreceptor" to "photoreceptors".

Corrected, thanks.

7. line 127, "we could still find STA with", suggest "we could still find a STA with".

Corrected, thanks.

8. line 128, as above, please explain how this accuracy in the diameter of the receptive field was determined based on the 67 um check size, and add a short section (maybe the same as for mouse, above) detailing the assumptions about receptive field shape.

We added one more comment on the Gaussian hypothesis in the method section (see before).

9. line 159, "which corresponds to repeat the flashes multiple times", suggest "which corresponded to repeating the flashes multiple times" or "which was equivalent to repeating the flashes multiple times."

Corrected, thanks.

10. line 185, "has not been performed", suggest, "was not performed".

Corrected, thanks.

11. line 188, "showed a small", suggest "had a small".

Corrected, thanks.

12. line 194, "larger light intensities", suggest "greater light intensities", or "brighter light intensities".

Corrected, thanks.

13. line 195, "cannot be stimulated", suggest "are not stimulated".

Corrected, thanks.

14. line 208, "but was never", suggest "but was not".

Corrected, thanks.

15. line 209, "optogenetics in a therapeutic purpose", suggest "optogenetics for a therapeutic purpose".

Corrected, thanks.

16. line 241, "one need to also", suggest "one needs also to".

Corrected, thanks.

17. line 271, "will transfected", suggest "will transfect".

Corrected, thanks.

18. line 385, "but limits the precision", suggest "but limited the precision".

Corrected, thanks.

19. line 395, "a Gaussian", suggest "Gaussian".

Corrected, thanks.

20. line 400, please explain "salient receptive field", or choose different wording, as the naive reader is confused by this phrase.

We now corrected this in "visible receptive field".

21. line 440, "enable to reach the light intensity", suggest "provide the light intensity".

Corrected, thanks.

22. line 446, " and therefore it will not", suggest "and therefore will not".

Corrected, thanks.

23. line 446, "therefore it will not affect our ideal Bayesian observer". I suggest that, strictly speaking, this is incorrect, as the ideal observer must take into account the variability in each cell by shifting ideal choices slightly away from the letter-independent noise distribution. Suggest "therefore it will minimally affect our ideal Bayesian observer" or similar wording.

Thanks, we now corrected this as "therefore it will minimally affect our ideal Bayesian observer"

24. lines 469-473, discussion of going from macaque to human: would be helpful here to mention the relative size of the macaque and human eyes, and how this would affect the comparison. The same size ganglion cel receptive field will give higher acuity in a larger eye (i.e. with a greater post-nodal distance).

We agree with the reviewer that the human eye is larger than the macaque eye and this could affect acuity for a receptive field of the same size: according to Dacey and Petersen (1992), one degree of visual angle corresponds to 275 microns in the human fovea, and 223 in the macaque fovea. However, the proper comparison should take into account both this factor and the relative size of midget cells in human and macaque retinas, by comparing dendritic field size converted in degrees of visual angle. This comparison was performed by Dacey and Petersen (1992) and they show that the sizes were very similar.
We have added this remark in the text.

Reviewer #3: The author clarified many concerns, added additional data, quantifications, and analyses and thereby strengthened the paper. However, I would have hoped that the authors take a more careful approach and further tune down their claims.

I doubt that the area of additional sensitivity in figure 1A is caused by 'random fluctuation of noise'. It might very well not be related to the axon, but it looks like what one might expect the RF of an initial axon segment to look like. Similar hints might be visible in figure S1 B and D. However, it is not strictly necessary to follow up on this.

We agree that we cannot tell if this receptive field corresponds to the soma or the axon initial segment. However, this does not change the conclusion of our results.

I hoped my comments on the model would result in more realistic versions in addition to the best-case scenario. Of course, the contrast in a prothese would be quite high, but possibly not permanently binary. While the current optimal Bayesian decoder is not affected by the rhythmic firing of unstimulated neurons, a decoder that uses the same likelihood, independently of whether a cell is stimulated or not, would be. This might better resemble the visual system of a patient prior to relearning.

We agree that our decoding analysis assumes that relearning has already occured. This has been added to the text (L211-212).

The description of the new analysis to estimate the error in the measured RF sizes due to coarse checkers is not clearly formulated. It seems that the authors doubled the background noise and refitted the Gaussian. I don't think that this is a good test. One possibility might be to subsample and then refit Gaussians of known size to simulate the effects of alignment and stimulus size.

We acknowledge that our text was not clear enough, and indeed we did something similar to what the referee is suggesting. After the Gaussian fit, we re-added Gaussian noise to the denoised (after fitting) STA and re-do the fitting. In this way the denoised Gaussian fit is first

subsampled to the checker scale and then corrupted by noise. Therefore the noise is not doubled. We rephrased the text to improve its clarity.
