# Supplementary material for: Towards optogenetic vision restoration with high resolution
Source: PLoS Comput Biol. 2020 Jul 15;16(7):e1007857. doi: 10.1371/journal.pcbi.1007857 (PMC7416966; doi:10.1371/journal.pcbi.1007857)
Supplement: Attachment — Submitted filename: PLOs Comp Biol.docx [file pcbi.1007857.s003.docx]

The manuscript by Ferrari et al. measures the receptive fields of optogenetically transdued retinal ganglion cells in mouse retina and in primate retina. Based on the spatial extend of the estimated receptive fields the authors build a model and theoretically estimate spatial acuity. The manuscript adresses a highly relevant topic, but the authors should give the paper the academic depth it deserves.

In the current version I cannot recommend the manuscript for publication. Please find my arguments below:

1. In the abstract the authors state: „..the spatial resolution of optogenetically-reactivated retinas has not been measured with fine-grained stimulation patterns.“

This statement is not correct, see i.e. the recent publications by (a) Barrett et al (2016 / <https://www.nature.com/articles/srep33565>) and by (b) Ganjawala TH et al. (2019). These studies investigated mouse retina only; however, the spatial resolution was measured in very great detail. This experimental scientific rigour is missing in the manuscript by Ferrari et al.

2. The authors measure the spatio-temporal receptive field of optogenetically transduced retinal ganglion cells using white-noise checkerboard stimuli.

This approach is not new either and has been reported recently by Pisano et al. (2017) <https://www.nature.com/articles/s41598-017-15741-7>. The authors should (a) cite this previous work and (b) explain if their results confirm / reject this (and pontetially other) work.

Furthermore, I am concerned that the temporal time course of the receptive field (Fig 1B) is wrong. In line 89 the authors state „ „The latency of the peak response was around 25 ms.“ This value is about ten times longer than direct activation of retinal ganglion cells in the mouse retina ! The peak response latency is not reported fort he trace presented in Fig. 2B.

3. The authors base their analysis for macaque retinal ganglion cells on 3 cells, i.e. those thre cells which already showed a high response relaibility ! This is a very low number and cetainly not enough for statistical comparison. Please include all 9 RGCs (line 303) recorded in the macaque retina.
4. The nonlinearity oft he LN model may be shown as well. It would be instructive to see if the the prediction performance (Fig. 1C&D; Fig. 2C&D) is different for linear estimates as compared tot he LN model.
5. The performance of predicting the stimulated response based on the LN model is not well explained. Please provide additional details (i.e. was there any temporal binning performed and if so at what resolution ?).
6. In the last part of the manuscript the authors build a model and use a Baysian decoder to predict the orientation of a presented stimulus („letter E“). Again, baysian decoding is not new and has been performed for optogeentically transduced retinal ganglion cells in the work of Barrett et al (2016 / <https://www.nature.com/articles/srep33565>). The in silico approach in this manuscript might be different but this difference needs to be explained and discussed in detail.

bstract:  it is academically shallow and reads more like a commercial motivator.   Though it is no problem that about half of the authors are potentially conflicted with industry, they should apply the same rigor to the abstract that they would to their other research publications. It currently reads as if written by a PR-agency than by scientists.  As it stands now, the abstract describes how novel the technique is and how much work went into it. But the abstract is missing reference to any statements regarding efficacy, safety, current challenges and future directions or a carefully balanced discussion about benefits and risks.

The interested reader wishes to know, for example, how many patients have benefits and what kind of benefits one can expect, for which cases it is useful, the long term consequence, and possible surgical or other risks. 

Minor revisions:

1. Figure Caption 1D: „ Prediction performance of the model against reliability of the response (see methods).

I do not see such a plot. Instead it seems as though average values are presented. Could you plot for each cell the reliability (x-axis) against prediction performance ? It would be informative to check for linearlity.

2. The visual stimulus is poorly described. I checked the cited reference (Deny et al. 2017), where exactly the same sentence is provided. „The stimulus was displayed using a Digital Mirror Device and focused on the photoreceptor plane using standard optics.“ Please provide the DMD supplier company and describe what optics has been used (microscope ?)
3. Line 168 – 171.The authors claim that no axonal stimulation occurs. This may be true on average (spike-triggered responses) but cannot be excluded by this approach. Additionally the authors seem to use only one light intensity, which could be below the axonal stimulation threshold. Please be more specific here.
